# Subtle Nanostructuring of the Au/Ru(0001) Surface

**DOI:** 10.1186/s11671-018-2611-5

**Published:** 2018-07-09

**Authors:** A. Goriachko, H. Over

**Affiliations:** 1 Department of Physical Electronics, Taras Shevchenko National University of Kyiv, Glushkova 4G, Kyiv, 03187 Ukraine; 2 Department of Physical Chemistry, Justus Liebig University, Heinrich Buff Ring 17, 35392 Giessen, Germany

**Keywords:** Gold, Ruthenium, Surface, Reconstruction, Scanning tunneling microscopy (STM)

## Abstract

We report on a scanning tunneling microscopy (STM) study of the nanostructuring of the Au/Ru(0001) thin film system for the cases of 5 monolayers (ML) and 9 ML of Au deposited at 300 K and subsequently annealed at 1050 K. A new laterally periodic superstructure is observed at the surface of the 9 ML film, which is essentially a rippling in height of the surface atomic layer with the magnitude up to 0.03 ± 0.01 nm and in-plane periodicity of 4.6 ± 0.4 nm, the long-range order being absent.

## Background

The Au(111) surface of bulk samples exhibits a rather unique 22 × √3 reconstruction as observed by STM [1, 2], which is now well understood in terms of atomic structure and electronic properties [3–6]. Normally, the Au(111)-22 × √3 reconstruction is explained by 23 atoms of the first surface layer sitting on top of the 22 atoms of the second layer, leading to orientationally degenerate contraction along the (110) direction. In order to minimize the free energy of the surface, the later splits into physically equivalent elastic stress domains of alternating orientation, which arrange themselves into a well-known herringbone pattern [7]. Obviously, a surface stress has a tremendous influence on the reconstruction of Au(111), so that one might expect its structural alterations if the surface stress varies. Indeed, it was found that single atomic steps release the tensile surface stress resulting in modifications to the herringbone pattern as a function of the terrace width [8, 9]. Additionally, the abovementioned pattern could have been modified locally amidst an atomically flat terrace by inducing local stress through artificially created surface defects by means of atomic manipulation with a scanning tunneling microscope tip [10]. Thin film samples of Au(111) can experience additional interface stress [11] due to lattice constant mismatch with the supporting substrate, again, influencing the subtleties of the surface reconstruction [12].

Our interest in thin film systems involving Au(111) stems from our previous work, where we observed an atomically flat surface of gold for a 14 monolayer (ML) film buried under a single layer of BN [13] and a 2 ML film [14], in both cases on top of the Ru(0001) substrate after annealing at 1050 K. Also, in the previous work of one of us, an atomically flat wetting layer was formed by 2 ML of Au deposited onto Ru(0001) at 700 K [15]. The flatness of the film surface at the atomic scale signals the possibility of the reconstruction, as intuitively expected for gold; however, there can be a departure from the standard (22 × √3-herringbone) picture due to additional stress, which is induced by the lattice mismatch between Ru(0001) and Au(111) characterized by in-plane lattice constants of 0.271 and 0.288 nm respectively. Indeed, a herringbone with unusually large period of about 100 nm was found for a 1 ML Au film and a distinctive trigon structure for a 2 ML film, both deposited on the Ru(0001) substrate at ~ 420 K and flash annealed at 790 K [16]. In the literature, one can also find investigations of the Au deposition on Ru(0001) at room temperature (RT), showing two-dimensional fractal or dendritic structures within the submonolayer films [17] and gradual nucleation and completion of subsequent atomic layers up to 3 ML coverage [18].

Evidently, the experiments reported in the literature mentioned above relate to the Au/Ru(0001) interface prepared in rather different temperature regimes, with an evident lack of information above the 3 ML thickness. Therefore, investigating thicker Au film on top of Ru(0001) was the goal of the present work. Here, we choose the following preparation scheme: deposition at RT and subsequent annealing at 1050 K — similar to our previous work.

## Methods

All experiments, including sample preparation and its characterization, were performed in a custom-built ultra-high vacuum (UHV) system; details have been described elsewhere [19]. The initial preparation of the single-crystal Ru(0001) substrate (sample size 5 mm × 5 mm × 5 mm, delivered by Mateck) consisted of sputtering with 1.5 keV Ar^+^ ions (Ar purity of 99.999%, delivered by Linde), the sample being kept at 1100 K to heal the damage to the crystalline structure of ruthenium. Next, the surface was exposed to molecular oxygen (purity 99.999%, delivered by Linde) at 5 × 10^−7^ mbar range for several dozen minutes, while keeping the same sample temperature. This treatment had removed carbon contamination from the near-surface region of the sample. Gold was evaporated onto the substrate at room temperature (RT) from Ø 0.25 mm wire (purity 99.99%, delivered by Sigma Aldrich) by an e-beam evaporator (delivered by Omicron) at a rate of 1 ML/min. The purity of our Au source was checked by means of Auger electron spectroscopy in a separate experimental setup, as well as calibrated by monitoring the Au (NVV, 69 eV)/Ru (MNN, 273 eV) peaks ratio. The surface topography of the samples was investigated in-situ by means of STM in constant current mode (VT-STM, delivered by Omicron). All measurements were performed at the background pressure in the UHV range and always after the sample has cooled to RT, the later in order to minimize a thermal drift and associated image distortions. We have used metallic probe tips hand cut from the Pt_80%_Ir_20%_ Ø 0.25 mm wire (purity 99.9%, delivered by Sigma Aldrich). These tips were conditioned in the tunneling regime by voltage and current pulses of the magnitude up to 10 V and 300 nA correspondingly, at surface locations far away from the actual imaging area. The pulses were applied until a stable imaging was possible at certain tunneling conditions, albeit different among different samples and experiments. A well-established (2 × 2)-O/Ru(0001) surface structure, featuring an easily resolved hexagonal array of O atoms with 0.54 nm lateral periodicity [20, 21], was used for calibration of our STM instrument. It was chosen because of the ease of its preparation in our experimental setup, essentially by a slight variation of the substrate preparation procedure. Namely, the oxygen exposure was terminated by turning off the sample heater while the oxygen supply was kept on for several minutes, leading to sample cooling in oxygen atmosphere. All STM data processing was performed using the Gwyddion software, which is freely available from the gwyddion.net website.

## Results and Discussion

**Fig. 1:**
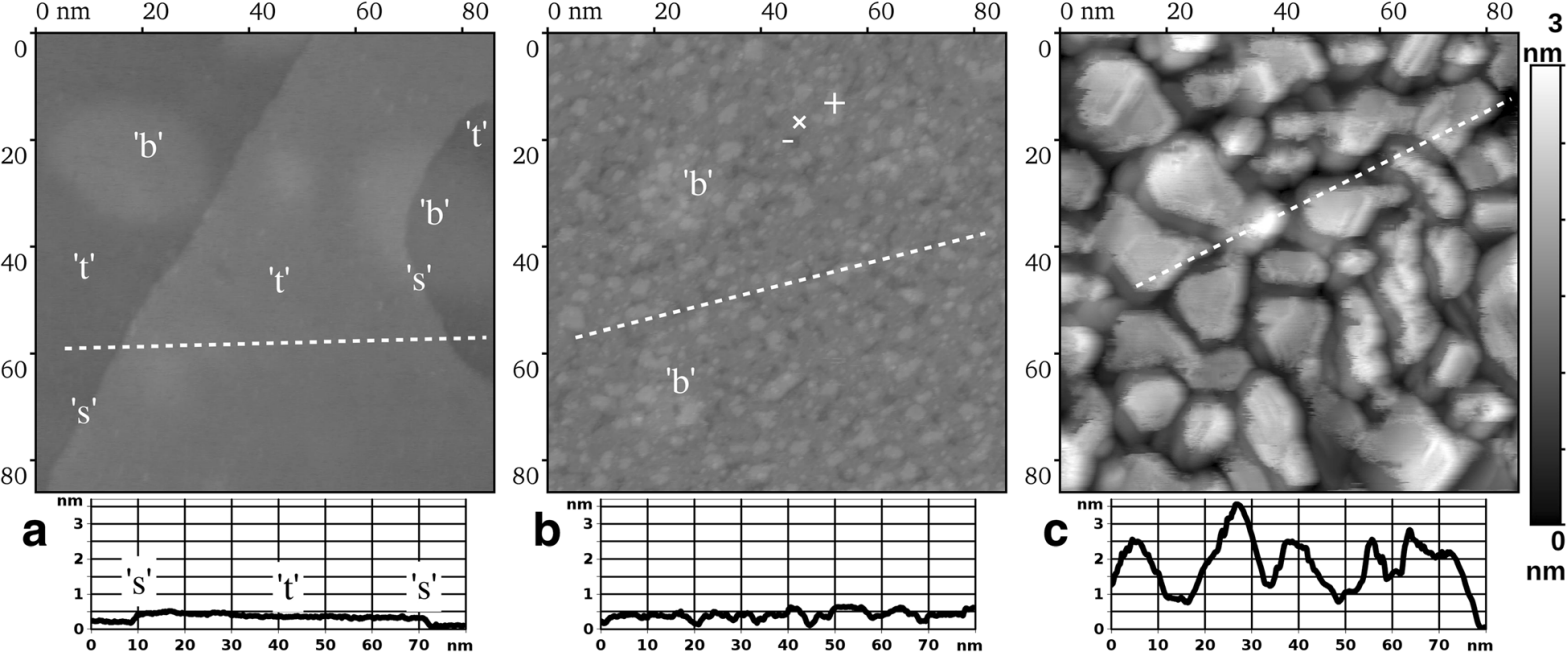
STM images (86 nm × 86 nm) of the single-crystal Ru(0001) sample at different stages of Au film growth: **a** the initial clean metallic substrate; sample bias voltage: *U* = + 0.1 V, tunneling current: *I* = 10 nA. **b** 5 ML Au film; *U* = − 0.05 V, *I* = 1 nA. **c** 9 ML Au film; *U* = 0.01 V, *I* = 1 nA. All images are presented in an identical gray scale (height-to-color correspondence), which is given rightmost. The height-distance cross-sections along the dashed lines are presented below every image. The meaning of designations within the images: “t” — atomically flat terraces, “s” — single atomic steps, “b” — locations above the buried argon bubbles, “×” — height level of the atomic layer corresponding to nominal coverage, “−” — one atomic layer below the nominal, “+” — one atomic layer above the nominal

First, we survey the surface morphology of Ru(0001) with and without the as-deposited Au film (see Fig. 1, STM images 86 nm × 86 nm) before annealing to 1050 K. In Fig. 1a, we observe a typical clean Ru(0001) surface resulting from our preparation procedure. It exposes atomically flat terraces “t” separated mostly by single atomic steps “s,” marked correspondingly both at the image and at the height-distance cross-section. On top of atomically flat terraces, we notice irregularly placed and shaped elevations “b,” which closely resemble elevations above the buried argon bubbles after similar preparation of Ru(0001) reported by Jakob et al. [22].

The case of as-deposited 5 ML film is presented in Fig. 1b. Essentially, we observe a roughening of the sample surface as a result of either Stranski-Krastanov or Volmer-Weber growth mode of Au on Ru(0001) at RT. It manifests itself by nucleation of some next atomic layer, while the previous atomic layer of the growing film is not yet complete. However, the Stranski-Krastanov and Volmer-Weber types of growth [23] can be differentiated on the basis of ref. [17], where the onset of the second layer nucleation was reported at 0.8 ML nominal Au coverage. Thus, our current data is in line with the Volmer-Weber growth mode in the Au/Ru(0001) system at RT. In Fig. 1b, we observe already three consecutive atomic layers of the adsorbate being simultaneously exposed to vacuum within the visible region of the sample—designated by the cross, plus, and minus signs. Keeping in mind the 5 ML coverage, one can tentatively assign them to fourth (“−”), fifth (“×”), and sixth (“+”) atomic layers of the growing Au film. Also, at this growth stage, one can still recognize the original surface locations above the buried argon bubbles, which are on average slightly brighter (higher) than their surroundings.

**Fig. 2:**
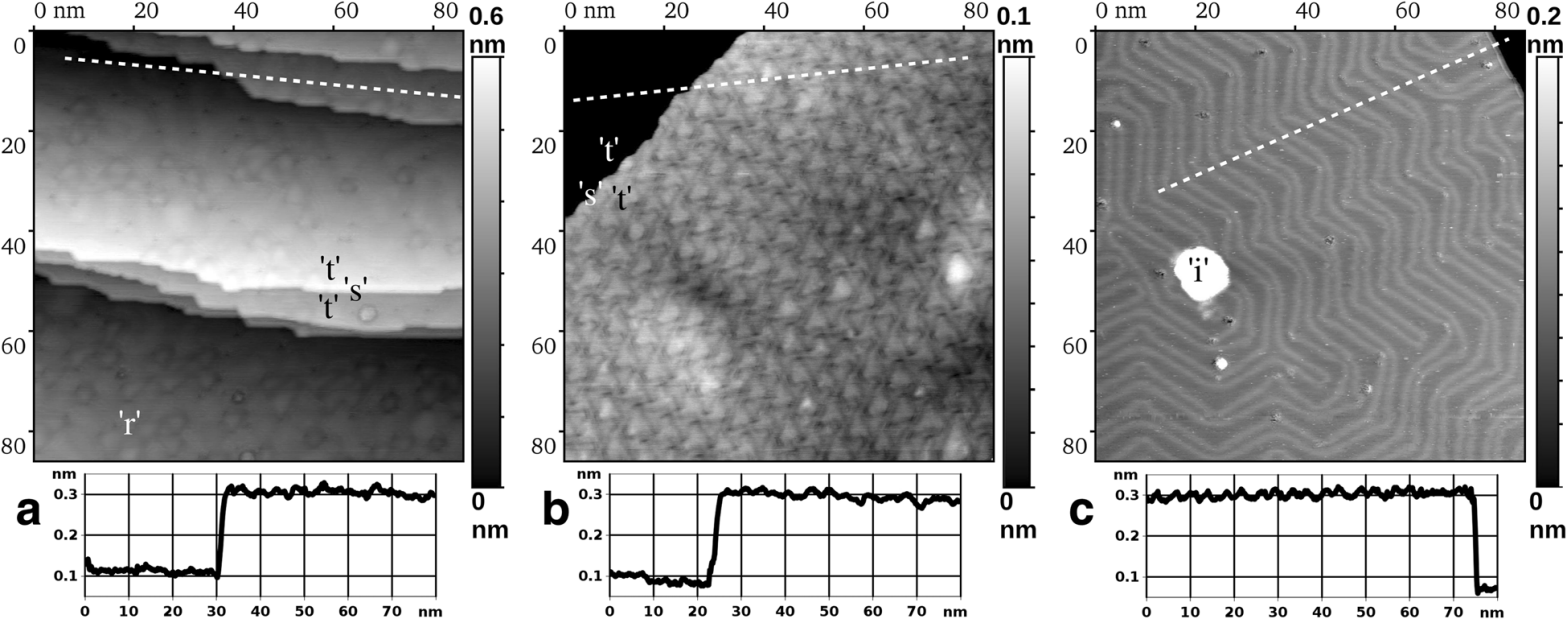
STM images (86 nm × 86 nm) of atomically flat Au(111) surfaces. **a, b** Thin Au films grown on Ru(0001) at RT and annealed at 1050 K during 5 min; **a** nominal coverage 5 ML, sample bias voltage: *U* = − 0.2 V, tunneling current: *I* = 3 nA, **b** 9 ML, *U* = − 0.003 V, *I* = 10 nA, **c** single-crystal Au(111) sample; *U* = − 0.003 V, *I* = 10 nA. Please note the different gray scales given to the right of every image. The height-distance cross-sections along the white dashed lines are presented below every image. Designations: “t” — atomically flat terraces, “s” — single atomic steps, “r” — ripples, “i” — impurity cluster

**Fig. 3:**
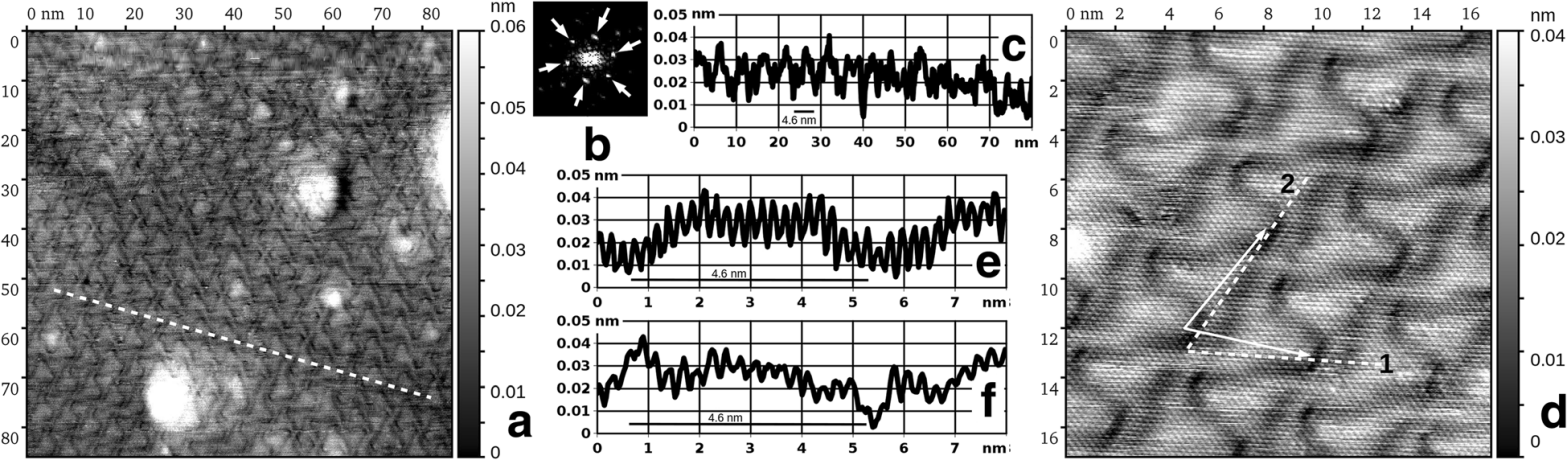
STM images of the 9 ML Au film grown on Ru(0001) at RT and annealed at 1050 K during 5 min: **a** field of view 86 nm × 86 nm, sample bias voltage: *U* = − 0.003 V, tunneling current: *I* = 10 nA. **b** FFT transform of the image (**a**) shown is a square section of the reciprocal space with a side of 1 nm^−1^, the 0th order spot is exactly in the middle. **c** Cross-section along the white dashed line in **a**. **d** Field of view 17 nm × 17 nm, sample bias voltage − 0.003 V, tunneling current 50 nA; white arrows designate the primitive translation vectors of the surface superstructure. **e, f** Cross-sections along the dashed lines 1 and 2 in **d**. An individual gray scale (height-to-color correspondence) is given to the right of images **a** and **d**. 4.6 nm bars are given as black solid lines on the graphs in **c**, **e**, **f**

Finally, in Fig. 1c, we present the highest amount of Au deposited on Ru(0001) in the present work, namely a 9 ML film. In this case, we observe a pronounced three-dimensional island structure. The Au film is essentially nanostructured in this state, while the lateral size of the islands is of the order of 10 nm. This is also accompanied by a substantial increase of the surface roughness, as can be concluded from comparison of all three cross-sections below the STM images in Fig. 1. Namely, in Fig. 1b, the magnitude of height variations of more than 3 nm indicates that more than 10 atomic layers are exposed to vacuum simultaneously. Thus, Fig. 1 illustrates the tendency of the Au growth on Ru(0001) at RT to proceed with a pronounced 3D islands formation at a late enough growth stage, whereas the surface of the sample departs far away from its initial atomic flatness. No subtle elevations due to “underground” Ar bubbles can be recognized on such a rough background. In Fig. 2a, b, we present the STM images (86 nm × 86 nm) of the same Au/Ru(0001) samples as in Fig. 1b, c but after additional annealing at 1050 K during 5 min in UHV. In both cases, we observe the surface consisting of atomically flat terraces “t” separated by single atomic steps “s,” as can be concluded from the cross-sections below the images. This means that our annealing procedure leads to ultimate smoothening of the as-deposited Au films. The case of 5 ML Au film is given by Fig. 2a. Here, within the terraces, we have consistently observed the rippling of the surface with the magnitude below 0.05 nm. The ripples “r” appear arbitrary in their shape and placement and do not form any ordered structure. The situation changes qualitatively in the case of 9 ML, namely in Fig. 2b, we observe the rippling of the same magnitude in height but with a highly regular ordering of the ripples, which are roughly triangular in shape. The thickness value of 9 ML is already large enough to approach the bulk properties of gold. Therefore, for the sake of comparison, in Fig. 2c, we show the STM image of the same size obtained on the single-crystal Au(111) sample. Its surface was prepared by a standard well established procedure of simultaneous ion sputtering and annealing, most of the surface being atomically flat apart from some small amount of impurity clusters “i.” Here, as expected, the flat terraces display a familiar “herringbone” pattern of the reconstructed Au(111), with a height modulation of similar magnitude as the rippling in Fig. 2a, b. The latter fact can be deduced from all three cross-sections in Fig. 2, each running across a single atomic step separating atomically flat terraces on its both sides. The surface structure in Fig. 2b deserves a special attention due to its regular nature and an obvious drastic difference from a single-crystal Au(111) surface structure. Since the presence of steps and neighboring terraces within a single image obscures the subtle height variations of any given atomically flat area, we have further investigated the same annealed 9 ML film, while choosing a location with a large enough terrace to fit into an STM image in its entirety. Such location is depicted in Fig. 3a with a field of view 86 nm × 86 nm, revealing certain irregularities in the pattern of surface rippling, as one can observe numerous abrupt changes in ripples’ ordering, as well as variations in their lateral periodicity, in other words – any long range order is absent in the given case. Additionally, this surface also displayed a certain amount of heterogeneities (areas with strong brightness variations), which could originate from impurities (on top or perhaps within the Au film) or subsurface argon bubbles (the latter could become discernible again, as the surface becomes mostly atomically flat as in Fig. 1a). In Fig. 3b, we present a fast Fourier transformation (FFT) pattern of the image in Fig. 3a, where the first order superstructure spots are clearly discernible (marked by white arrows). Converting their distance from the (0,0) spot into the real space periodicity gives three values of 4.44, 4.76, and 4.55 nm, which are rather close to each other and hint on the hexagonal unit cell distorted by thermal drift, piezo creep, and other known artifacts of the STM technique. However, an oblique unit cell of the superstructure cannot be excluded in our study. The average of these three values, that is ~ 4.6 ± 0.4 nm, is the best current estimate of the periodicity of Au surface rippling in the (9 ML Au)/Ru(0001) film/substrate system after attaining thermal equilibrium during annealing at 1050 K. Here, the range where the actual periodicity values are scattered was obtained from the half-width of the FFT first order spot. The cross-section in Fig. 3c was obtained along the white dashed line in Fig. 3a, which avoids any surface heterogeneities. It shows the magnitude of the rippling of the order of 0.02 nm; however, using it to measure the lateral periodicity of the superstructure may be misleading due to artifacts mentioned above.

Finally, in Fig. 3d, we observe a small surface area (17 nm × 17 nm) containing several superstructure unit cells, which can be considered laterally periodic at this scale. This image was obtained with atomic resolution, so the cross-sections in Fig. 3e, f were obtained along high-symmetry directions of the atomic lattice (white dashed lines 1 and 2). The magnitude of the height corrugation between the individual atoms is typically within the range from 0.005 to 0.015 nm, while the magnitude of the surface rippling is roughly 0.03 nm, slightly higher than in Fig. 3a (which may be explained by a higher setting of the constant tunneling current). Therefore, based on available data, the best estimate of the uncertainty of the measured surface rippling is ± 0.01 nm. We were reluctant to extract the exact interatomic distance within the topmost layer from cross-sections (3e, f), due to STM artifacts already mentioned above, pending a dedicated investigation by means of diffraction techniques. White arrows outline the sides of a unit cell of the superstructure arising due to surface rippling. In the given location, its lateral periodicity is roughly 5 nm, which is somewhat larger than the average value obtained by FFT from Fig. 3a. An important observation is a directional non-coincidence of the superstructure’s translation vectors and the high symmetry directions of the atomic lattice. Further, this angular deviation is different for both of these vectors, which may indicate the first and the second surface layer being rotated relative to one another. Again, the exact angular values could not be extracted due to lateral distortions within the image. If the true periodicities along the dashed lines 1 and 2 are different (meaning an oblique unit cell of the surface atomic lattice), then there is an anisotropic contraction of the topmost atomic layer, which is also the case for the standard Au(111)-22 × √3 reconstruction. On single-crystal Au(111), the resulting stress is released through spontaneous formation of the herringbone superstructure, while in the case of Figs. 2b and 3a, it is the absence of the long-range order, which will be equivalent to spontaneous formation of a set of orientation-degenerate elastic strain domains.

The superstructure in Fig. 3d resembles the trigon structure reported by Ling et al. for the 2 ML Au film on Ru(0001) [16]; however, a precise examination of the corresponding STM images reveals that they are not identical. They are also very different by the nature of their preparation: deposition at ~ 420 K and flash annealing at 790 K for the trigon structure [16] as opposed to RT deposition and prolonged annealing at 1050 K in the present work. Clearly, all these structures, including a disordered surface rippling on top of the 5 ML in Fig. 2a, result from different stress experienced by the Au film. However, caution is advised in relating a certain film thickness to the superstructure observed on its surface, as differences in thermal treatment may result in different structures with different stress values even for the same nominal thickness. Although Au and Ru do not form bulk alloys [24, 25], there is experimental evidence that surface alloys can be formed in this system [26]. We speculate that the degree of such alloying can be influenced by the temperature and duration of thermal treatment, resulting in the strained Au film with a lattice constant anywhere from bulk Ru to bulk Au values. This uncertainty prevents us from trying to build a tentative atomic model of the new superstructure depicted in Figs. 2b and 3a,d. This can be realistically performed only knowing the precise actual values of the lattice constants in both the first and the second atomic layers, which can be obtained from diffraction experiments. In parallel, more precise STM measurements should be performed with thermal drift correction being applied in order to increase the accuracy of the obtained real-space data on the first atomic layer.

Further experiments are also required to further elucidate the thickness dependence of the nanostructuring pattern. The most intriguing question if the bulk-like herringbone pattern will be achieved at high enough thickness values. The data available so far show three qualitatively different cases (for our preparation route): no nanostructuring up to 3 ML Au, unordered rippling at 5 ML, and ordered rippling of the surface of the 9 ML film. Therefore, our preliminary experiments reported in this paper confirm our initial hypothesis that the varying film thickness will lead to different reconstructions of the Au(111) surface in the Au/Ru(0001) system. They hint on some intricate dependence of the nanostructuring on the Au film thickness, thus warranting further detailed studies with more different amounts of deposited material. Additional effort will be required to avoid any possible instrumental artifacts or uncertainties, in particular, obtaining all the STM images in identical tunneling conditions (this will require more attempts to prepare the probe tips, which produce stable tunneling current at the same bias voltage on different samples).

Any possible applications of the new superstructure would be roughly of the same practical value as that of the Au(111) herringbone self-assembled nanoscopic pattern (keeping in mind traditionally high cost of the single-crystal metal substrates). The latter is a proven nanotemplate for creating highly regular molecular arrays by exploiting preferential adsorbtion of suitable molecules in certain parts of the surface unit cell. In a similar manner, the newly found 4.6 nm superstructure may find uses as a nanotemplate for molecular arrays but of lateral periodicity and symmetry different from that on single-crystal Au(111).

## Conclusions

In conclusion, we have identified by means of STM investigation both disordered and ordered rippling of the surface of Au(111) film on top of Ru(0001) substrate for 5 ML and 9 ML nominal thickness, respectively. In the latter case, a hexagonal or oblique superstructure is formed with an average in-plane periodicity of 4.6 ± 0.4 nm but with no long-range order. It is believed that this rippling is similar in nature to the well-known Au(111)-22 × √3 herringbone reconstruction observed on single-crystal samples of gold. The exact rippling pattern of the newly reported superstructure results from the interplay of different interatomic distances on the surface and inside of the Au film, which are not yet precisely established. Further investigations with various diffraction techniques as well as ab-initio modeling would be required in order to establish an exact atomic model of the reported surface superstructure.

## Abbreviations

ML: Monolayer
RT: Room temperature
STM: Scanning tunneling microscopy
UHV: Ultra-high vacuum
